# Influence of Personality Traits on the Severity of Alcohol Use Disorders

**DOI:** 10.3390/jcm7060127

**Published:** 2018-05-29

**Authors:** Aida Álvarez, José J. Ávila, Diego J. Palao, Ángel L. Montejo

**Affiliations:** 1 Department of Psychiatry, Corporaciò Sanitária Parc Taulí, 08208 Sabadell, Spain; dpalao@tauli.cat; 2 EUEF School, University of Salamanca, 37008 Salamanca, Spain; avilaescribano@gmail.com (J.J.Á.); amontejo@usal.es (Á.L.M.); 3 Centro de Investigación en Salud Mental (CIBERSAM), 28029 Madrid, Spain; 4 Institute of Biomedical Research of Salamanca (IBSAL), 37007 Salamanca, Spain; 5 Departament of Psychiatry, University Clinical Hospital of Salamanca, 37007 Salamanca, Spain

**Keywords:** alcohol disorder, personality, severity, cloninger

## Abstract

Introduction: Alcohol Use Disorders (AUD) are the most prevalent psychiatric diagnosis in the general population. The study of personality characteristics, using Cloninger Personality Inventory (TCI-R), allows us to know the evolution of these patients at the beginning of treatment. Material and Method: We conducted a cross-sectional, observational and descriptive study for 3 years with a total of 304 patients. We studied the severity of their alcohol disorder by the Alcohol Dependency Intensity Scale (EIDA), Scale of Obsessive Consumption Compulsive (OCDS) and European version of the Addiction Severity Index (EUROPASI); we studied the relationship with the personality traits of TCI-R. Results and conclusions: The personality lines influence the evolution of alcohol use disorder (AUD). People with higher scores on Reward Dependency (RD), Persistence (P), Cooperation (CO) and Autotranscendence (ST) have a better prognosis while people with higher scores on Search for Novelty (SN) and Avoidance of Damage (AD) have a worst prognosis. Women present differences in consumption in relation to men, as a consequence of their personality. Women have lower scores in Persistence (P) y Self-Transcendence (ST) which are associated with the greater severity of their addiction.

## 1. Introduction

Alcohol use disorders (AUD) are the most prevalent psychiatric diagnosis in the general population. It is estimated that in Spain, around 7–10% of the adult population (3 million people) has problems related to alcohol consumption. This constitutes a high relevant public health problem [1,2,3].

Multiples studies have analyzed personality characteristics in patients with an AUD. The vast majority of studies conducted in patients with addiction problems, in order to know their personality traits, have been made through the Cloninger Temperament and Character Inventory (TCI), based on his psychobiological personality model (Cloninger, 1987) [4]. For Cloninger, the personality reflects differences in the responses of neuroadaptive systems related to learning mediating two concepts mainly: the concept of temperament and the concept of character. The temperament is defined as the individual differences in habits and abilities related to emotions, is inherited around 50%. Four features are described: Search for Novelty (BN), Avoidance of Damage (AD), Dependence on Reward (DR) and Persistence (P). The character of the personality is inherited to a lesser degree and is moderately influenced by social learning. There are three characteristic dimensions: self-direction (SD), cooperation (CO) and self-transcendence (ST) [1,2,3].

Differents studies found higher scores in people with AUD than in the general population, in terms of disinhibition, impulsivity, antisocial behaviour and aggressive personality. These features suggest low control capacity [5,6]. If we focus on the variables quantified by the Cloninger Personality Inventory (TCI-R), then the results are not as conclusive. Some authors report high scores in Avoidance of Damage (AD) [7], while others observe high scores in Search for Novelty (SN) and Avoidance of Damage with low scores in Self-Direction (SD) [8]. However, there are few studies that relate the personality traits of patients with the severity of their psychiatric pathology. The early detection of at-risk drinkers is a priority from a public health perspective because of the high prevalence of risk consumption and the effectiveness of treatment aimed at reducing consumption.

The main objective of this study doctoral thesis study was to investigate how—and to what extent—personality traits, at begining of the treatment, influence the severity of AUD and what differences exist according to gender [9].

## 2. Materials and Methods

This is a cross-sectional, observational and descriptive study carried out in the Alcoholism Treatment Unit of the Hospital Clínico Universitario de Salamanca with duration period of 3 years (from 2013 to 2016) including a sample of 304 patients patients (describir % hombres y mujeres y edad media con DS). The sample is composed of 304 patients affected by Alcohol Use Disorder (AUD), who attended the Alcohol Disorders Unit of Salamanca, of which 250 were men (82.2%), and 54 women (17.8%). The average age of men was 43.6 years (SD 9.7); and in the case of women of 43.8 (SD 9.8).

Regarding the diagnosis, 244 patients (80.3%) of the total sample fulfilled alcohol dependence criteria, whereas 60 (19.7%), suffered from an alcohol abuse disorder. 78.9% of men (197) had an alcohol dependence, while 21.2% (53 men) had an abuse disorder. In the case of women, 87.0% (47 of them), had a diagnosis of alcohol dependence, compared to 13.0% women (7), which presented a pattern of abuse, although these differences were not significant.

Patients had been diagnosed with an AUD and were between 18 and 70 years of age and of both sexes. They collaborated voluntarily and signed an Informed Consent. The study was approved by the Ethical Committee of the Clinical Hospital of Salamanca. Patients with concomitant consumption of other substances, with cognitive difficulties, illiteracy, under 18 years of age and with comorbidity with other serious mental disorders were excluded from the study.

Through the semi-structured interview, relevant data were collected, as follows: sociodemographic data, consumption pattern, consumption characteristics, type of alcoholic beverage and grams of alcohol consumed. In addition, the clinical course of the disorder, the diagnosis of AUD and comorbid entities were assessed. Finally the degree of motivation for the treatment and its type were also registered. The rest of the instrumental tests were then executed.

1. EUROPASI scale (European version of the Addiction Severity Index) [10,11]: This basic instrument for clinical practice allows a multidimensional diagnosis of addiction problems. It assesses their severity and puts them in a bio-psycho and social context. This instrument evaluates different aspects of the lives of patients affected by an addictive pathology. Specifically, it explores the following six potentially problematic areas of life: Physical Health (16 items); Employment/Resources (26 items); Drugs/Alcohol (28 items); Legal situation (23 items); Family history (51 items); Family/Social Relations (26 items); Mental Health (22 items).
2. EIDA Scale (Alcohol Dependency Intensity Scale) [12]: This is a self-applied measuring instrument that is composed of subscales: physical symptoms of abstinence, psychological symptoms of withdrawal, behaviours to relieve withdrawal symptoms, etc. It distinguishes between mild, moderate and severe dependence.
3. Questionnaire on the obsessive-compulsive components of drinking (OCDS) [13]: This tool evaluates the intensity of the components obsessive (preoccupation with drinking) and compulsive nature of the drink. According to this model, both components have their common base in desire (“craving”). This questionnaire addresses alcoholism under the model of an obsessive-compulsive disorder.
4. TCI-R questionnaire. The Cloninger Temperament and Character Inventory revised version (TCI-R) [14] The vast majority of studies conducted in patients with addictive behaviours and their personality traits, have been made by using the Cloninger Temperament and Character Inventory (TCI), (Cloninger, CR; Svrakic, DM ; Przybeck, TR 1993) [14], which is based on his psychobiological personality model (Cloninger, 1987). For Cloninger, the personality reflects differences in the responses of neuroadaptive systems (Cloninger, CR 1994, Cloninger, CR, Przybeck, TR, Svrakic, DM, Wetzel, RD 1994, Cloninger, CR 1987, Cloninger, CR; Svrakic, DM, Przybeck, TR 1993). Two main concepts arise from this approach: the concept of temperament and the concept of character.
  The temperament of the personality is defined as the individual differences in habits and abilities related to emotions. It is inherited around 50%, moderately stable from childhood to adulthood, and it seems to be consistent in its structure in different cultures and ethnic groups. Four features or temperamental dimensions are described: search for novelty (BN), avoidance of harm (ED), dependence on reward (DR) and persistence (P).
  - Search for Novelty (SN): hereditary tendency towards the search for excitement and interest in novel stimuli. This trait is mediated by dopamine. These people would be impulsive, excitable, curious and enthusiastic.
  - Avoidance of Damage (AD): this is the hereditary tendency to respond intensely to signs of adverse stimuli stimulating the system of behavioral inhibition, probably through serotonin. Subjects with high values in this dimension develop conditioned avoidance responses to aversive stimuli, which makes them cautious, apprehensive and fearful.
  - Dependence of Reward (DR): corresponds to the tendency to respond intensely to reward stimuli or signals and to maintain the behavior previously associated with said reward. This response is mediated by norepinephrine in the maintenance system or behavioral persistence. It has to do with social reinforcement and sensitivity to social stimuli and discomfort for the separation of the group.
  - Persistence (P): which implies a neurobiological tendency to maintain behaviors under conditions of extinction. That is, the ability of the body to continue emitting behavior associated with reinforcement despite the disappearance of it. It would be associated with serotonergic transmission and orbitofrontal circuits.

The character or conceptual core of the personality is inherited to a lesser degree and is moderately influenced by social learning and cultural expectations about the social role in relation to age, occupation and other social circumstances (Cloninger, CR, Svrakic, DM; Przybeck, TR 1993) [13]. There are three characteristic dimensions: self-direction (AD), cooperation (CO) and self-transcendence (AT)

- Self-direction: it is about the ability to regulate behavior and to commit oneself to the chosen goals
- Cooperation: is the ability to identify and accept others. It refers to the prosocial behavior of the subject. It has to do with altruism, empathy and solidarity
- Self-transcendence: represents the ability to identify as an integral part of the universe. It is related to creativity, fantasy and imagination (Bordalejo, D., Boullosa, O., Hadid, E., Puricell, M., Romero, E., Tannenhaus, L., Vieitez, A., Vázquez, G.; 2014 [15]).

The staircase consists of 240 elements that respond on a Likert scale of 5 options: 1 = False/2 = Almost all false/3 = True true than false/4 = Almost true/5 = True. Each of the dimensions of temperament and character is punctuated, according to a variable number of subscales, between 3 and 5.

For the TCI inventory, the revised version in Spanish (TCI-R) of Bayón (2004) was used and validated in our country by Gutiérrez-Zotes et al. (2004) In this version, the score of each trait is transformed into a “T” score (percentiles). Wong et al. (2010) define prominent features found in the upper third (67th percentile) or in the lower third (33th percentile) [15].

Statistical analysis used descriptive statistics, bivariate analyses and multivariate analysis.

Descriptive statistics: In all cases, the normality of the variables was verified with the Kolmogorov-Smirnov test. The quantitative variables were expressed as mean ± standard deviation and the qualitative variables were expressed as number and percentage.

Bivariate analyses: The Chi Square Test was used to analyze the association between quantitative variables. Using Student’s *t*-test or U Mann Whitney, according to the normality of the variables. To analyze the relationship between quantitative variables, the Pearson or Spearman correlation was used according to the normality of each variable.

Multivariate analysis: In the multivariate analysis, multiple linear regression analysis was used, using the multivariate linear general model (GLM), to analyze the relationship of personality with severity, intensity and the obsessive-compulsive component of consumption adjusted for age and sex.

In all cases, hypothesis testing used an alpha risk of 0.05 as the limit of statistical significance via IBM Statistical Package for the Social Sciences (SPSS) version 23.0 (IBM Corp., Armonk, NY, USA).

## 3. Results

The sample consisted of 304 patients affected by Alcohol Use Disorder (AUD); 250 were male (82.2%) and 54 were female (17.8%). The average age of the men was 43.6 years (SD 9.7) and 43.8 (SD 9.8) for women. Most patients came from an urban environment (193 (63.5%)), while 111 patients (36.5%) came from a rural setting. Here, 163 (53.6%) had a partner, 86 (28.4%) were single and 54 (17.8%) were separated.

**Figure 1:**
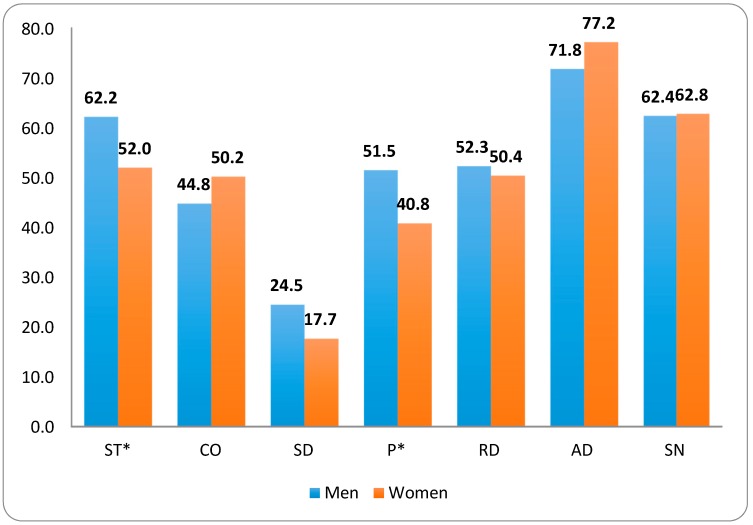
Cloninger’s (TCI-R) personality scores as a function of gender (P* and ST* statistically significant).

This cohort had high scores for Search for Novelty and Avoidance of Damage and a very low score in SD. According to Cloninger, this low score is associated with a personality disorder. Given that women represent only 17.8% of our study sample, we considered it relevant to analyse personality characteristics as independent samples according to sex. The women had significantly lower scores in Persistence (*p* = 0.03) and Self-Autotranscendence (*p* = 0.02) (Figure 1).

The temperamental traits of Search for Novelty (SN) and Avoidance of Damage (AD) correspond to a higher degree of AUD severity. That is, people who are more impulsive and curious and/or more pessimistic and fearful of change present a greater severity of their AUD. They start consuming alcohol at a younger age and tend to do some in a pathological way. This consumption occurs with greater intensity and with serious dependence. In addition, this group of subjects has a greater compulsion and desire (“craving”) for the drink, which produces a greater medical severity (Table 1 and Table 2).

However, the high scores in Reward Dependency (RD) and Persistence (P) are related to a lower severity of the disorder. In other words, people who were more sociable, ambitious and perfectionist had less intensity of dependence, less psychiatric severity and less craving for the substance. This translates into a less severe AUD (Table 3 and Table 4).

On the other hand, characteristic features, Self-Direction (SD), Cooperation (CO) and Self-Transcendence (ST) are unanimously related to a lower severity of the disorder by alcohol consumption. There is a lower intensity of dependency, less craving for drinking and less severe work and psychiatric severity (Table 5, Table 6 and Table 7).

Gender influences the severity of the alcohol consumption disorder via personality according to Cloninger. That is, three of the four temperamental variables (Search for Novelty, Avoidance of Damage, and Reward Dependency) are influenced by sex in their relationship with the severity of the AUD. This is seen through the duration of pathological consumption, medical severity, type of dependence, intensity of consumption, and objective family severity. On the other hand, SD and Self-Autotranscendence are influenced by sex in their relationship with the severity of the TCA through the obsession with drinking, the desire (“craving”) for it, and through psychiatric severity.

This means that the women in our study had significantly lower scores than men in Self-Autotranscendence and Persistence, i.e., they were less creative, spiritual, and ambitious than men—this translates into a greater severity of their addiction through a longer duration of pathological consumption, greater intensity of alcohol dependence, greater family and psychiatric severity, greater obsession and greater craving than drinking men (Table 8).

## 4. Discussion

Through Through this study, we wanted to predict the severity of AUD by means of the assessment of their personality at the initial phase of treatment. This approach would allow us to know, at the initial stages of treatment, how many patients may probably have a more severe clinical course, how many patients would suffer from subsequent higher number of relapses and more alcohol consumption, greater craving and greater severity of dependence. The aim is to be able to optimize resources at the beginning of treatment. In our study we found that our AUD cohort had high scores SN, AD and SD. That is, AUD patients suffer from more impulsive personality, show a tendency to experience new emotions (SN) or are more fearful and apprehensive (AD). They may also have a lack of objectives or attribute their problems to others (SD) (Wong et al., 2010). These results are in line with some other authors who have studied the personality characteristics of these patients in similar settings [16,17,18,19,20].

Next we will expose the results we found between personality and severity of the AUD that we have found: 

The scores described here are related to a higher severity of the AUD. The positive correlation of the Search for Novelty and Avoidance of Damage dimensions show a more severe dependence on alcohol (EIDA) and greater obsession and compulsion for drinking. These features indicate the relationship of these personality traits with a higher severity of AUD similar to other studies [18,19]. Nöel et al. (2011) concluded that alcohol-dependent subjects with high Search for Novelty scores have worse evolution and greater AUD severity and are more prone to relapse [8]. This may be due to the need of these people to experience new emotions even though this fact entails a risk to their physical and/or mental health.

The low score in Self-Direction observed here has been related to a greater severity of AUD through a more severe dependence on alcohol (EIDA) and more psychiatric pathology. On the other hand, high scores on this item are a protective factor—patients with a greater sense of responsibility and discipline have less severe AUD. Other studies have found similar results [20].

We conclude that people with high scores in Reward Dependency and Persistence as well as in Cooperation and Self-transcendence have less severe AUD. That is, people who are more social, hardworking, ambitious, empathetic and creative have a better prognosis in their AUD. Different authors corroborate our results and show that patients with higher scores in Persistence and Cooperation have a better evolution and less seriousness in their AUD [20,21,22,23,24].

The severity of alcohol dependence is determined by different variables, which show us statistically significant results if we analyze the sample according to gender. In relation to the severity of alcohol dependence by means of the EuropAsi scale, we find statistically significant gender differences. Women start drinking alcohol later compared to men (18 years vs. 15.6 years), this becomes problematic at older age (30.3 years vs. 22.9 years), the duration of this problematic consumption is lower (13.4 years vs. 20.3 years) and drink less alcohol (113 gr vs. 144), all of these differences being statistically significant (*p* = 0.001). Although women drink for fewer years and consume less alcohol, we find that women’s addiction is more severe when atteding to some other variables:

- The total score of the EIDA in our sample is 27.79 (SD 15.0), being 27.0 (SD 14.4), in the case of men; and of 32.5 (SD 16.7), in the case of women. Therefore, alcohol dependence is significantly more severe in women than in men (*p* = 0.01).
- In relation to the OCDS scale, the total score of the sample is 15.1 (SD 7.8). Men obtained a score of 14.6 (SD 7.2) and women 17.4 (SD 10.0), that is, women reported significantly more desire for alcohol than men. The average score on the obsession subscale is 6.3 (SD 4.1); in men the mean is 6.0 (SD 3.9) and in women 7.6 (SD 5.0), a very significant difference (*p* = 0.008). On the other hand, the scores obtained in the compulsion subscale would be 8.87 for the total sample (SD 4.4); being in the case of men of 8.7 (SD 4.1) and in the case of women of 9.8 (SD 5.4). In conclusion, women have a greater obsession and compulsion for drinking than men in a significant way.

These scores are related to a worse evolution and a higher number of relapses, showing greater difficulty in maintaining abstinence (Pedrero et al., 2006) [25].

It is widely known that most of the addictive disorders are more frequent in men than in women. Particularly, in our study, 82.2% of the sample were men, whereas 17.8% were women. Despite this, women, who start drinking alcohol later and drink less alcohol than men, have a higher severity of their disorder, greater dependence on drinking and greater craving for alcohol, as exposed previously.

If we correlate these results with personality, we find that women show higher scores in Self-Transcendente and Persistence, which implies less ambition and/or greater difficulty in maintaining behaviors without positive reinforcement. They also present lower scores in Self-Transcendence, which is associated with less creative and intuitive people, these findings could be related to the fact that most of the women in our study do not perform paid work (25.9% vs. 22% of men), or are dedicated to the care sector (27.8% compared to 0.8% of men). In addition, there is a smaller number of women with both superior and elementary studies than men, which is justified in part, with the social reality in Spain to date, in which women have been relegated to a lower socio-economic status.

This study is limited in that it is an observational, naturalistic, epidemiological and usual clinical practice study; the sample was recruited in a non-experimental way. Enrolling more subjects would better approximate reality. Another limitation is that we used the TCI-R inventory that consists of 240 items. In some cases patients could not complete it due to lack of time or due to difficulties in compression. This could be solved via the abbreviated versions with 140 (Pedrero, 2006; Wedekind, 2013) [25,26] or 60 items (Pedrero, 2009) [27] although these are in the experimentation and validation phase. Finally, the gender differences do not follow a homogeneous distribution in our sample, following a naturalistic method in a real world setting.

By means of the present research work we were able to know the probable evolution of the AUD in the first visit of our patients, which allows us to carry out more specific interventions from the beginning oriented to avoid a bad evolution in the patients more serious and to optimize resources.

Future work will account for the information obtained about the personality profile of patients with greater severity of AUD. This information is only descriptive, and it is ideally suited for controlled designs that are different from this study. The effectiveness of a more targeted intervention could be assessed in those patients with a more serious profile.

## 5. Conclusions

The severity of AUD, in our study, is mainly given by the EUROPASI, EIDA, and OCDS scale. These are used to diagnose alcohol abuse and/or dependence. There were correlations between personality and the different variables. High Search for Novelty and Avoidance of Damage scores are related to a greater TCA severity, which is defined by an earlier age of onset in consumption, greater dependence on the substance (higher score of EIDA), and greater obsession and compulsion for drinking as well as a greater total score on the OCDS scale. High scores for Reward Dependency, Persistence, Self-Direction, Cooperation, and Self-Transcendence are associated with a lower severity of AUD and better prognosis. This is seen via a lower intensity of dependence (total EIDA), less obsession with the substance, and less psychiatric severity. The women in our study have a more severe AUD than men. They were less creative (Self-Transcendence) and ambitious (Persistence). This would imply that they had a greater dependence and “craving” for the drink than men do.

## Figures and Tables

**Table 1:** Statistically significant correlations between Search for Novelty (TCI-R) and severity factors of the EIDA, OCDS and EuropAsi scales. (* *p* < 0.05; ** *p* < 0.001).

	Search for Novelty	*p*
Age at which consumption began	−0.263 **	<0.01
Age when consumption became pathological	−0.0207 **	<0.01
Grams of alcohol	0.131 *	<0.05
Total EIDA score	0.190 **	<0.01
Obsessive component score (OCDS)	0.116 *	<0.05
Compulsive component score (OCDS)	0.119 *	<0.05
Total OCDS score	0.123 *	<0.05

**Table 2:** Statistically significant correlations between Avoidance of Damage (TCI-R) and severity factors of the EIDA, OCDS and EuropAsi scales. (* *p* < 0.05; ** *p* < 0.001).

	Avoidance of Damage	*p*
Psychiatric Severity	0.225 **	<0.01
Alcohol Severity	0.126 **	<0.01
Age started consumption	0.118 *	<0.05
Diagnosis	Dependence	<0.05
Serious Dependence	0.306 **	<0.01
Total EIDA score	0.176 **	<0.01
Obsessive Component Score (OCDS)	0.189 **	<0.01
Compulsive Component Score (OCDS)	0.207 **	<0.01
Total OCDS Score	0.220 **	<0.01

**Table 3:** Statistically significant correlations between Reward Dependency (TCI-R) and severity factors of the EIDA, OCDS and EuropAsi scales. (* *p* < 0.05; ** *p* < 0.001).

	Reward Dependency	*p*
Psychiatric Severity	0.170 **	<0.01
Age when consumption became pathological	0.136 *	<0.05
Duration of consumption	0.114 *	<0.05
Total EIDA Score	−0.126 **	<0.01

**Table 4:** Statistically significant correlations between Persistence (TCI-R) and severity factors of the EIDA, OCDS and EuropAsi Scales. (* *p* < 0.05).

	Persistence	*p*
Psychiatric Severity	−0.139 *	<0.05
Compulsive component score (OCDS)	−0.145 *	<0.05
Total OCDS score	−0.123 *	<0.05

**Table 5:** Statistically significant correlations between SD (TCI-R) and severity factors of the EIDA, OCDS and EuropAsi scales. (* *p* < 0.05; ** *p* < 0.001).

	Self-Direction	*p*
Psychiatric Severity	−0.152 **	<0.01
Diagnosis	Dependence	<0.01
Serious Dependence	−0.243 *	<0.05
Total EIDA score	−0.321 **	<0.01
Obsessive component score (OCDS)	−0.359 **	<0.01
Compulsive component score (OCDS)	−0.295 **	<0.01
Total OCDS Score	−0.352 **	<0.01

**Table 6:** Statistically significant correlations between Cooperation (TCI-R) and severity factors of the EIDA, OCDS and EuropAsi scales^.^ (* *p* < 0.05; ** *p* < 0.001).

	Cooperation	*p*
Age when consumption became pathological	−0.163 *	<0.05
Duration of consumption	0.22 *	<0.05
Labour severity	−0.129 *	<0.05
Total EIDA score	−0.171 **	<0.01
Obsessive component score (OCDS)	−0.119 *	<0.05
Compulsive component score (OCDS)	−0.163 **	<0.01
Total OCDS score	−0.158 **	<0.01

**Table 7:** Statistically significant correlations between Self-Transcendence (TCI-R) and severity factors of the EIDA, OCDS and EuropAsi scales. (** *p* < 0.001).

	Self-Transcendence	*p*
Psychiatric Severity	−0.120 **	<0.01

**Table 8:** Relationship of personality with the severity of the AUD accoing to Cloninger. This was influenced by gender in a statistically significant way.

	Gender–Search for Novelty	*p*
Duration of pathological consumption	0.029	<0.05
Total EIDA score	0.041	<0.05
	Gender—Avoidance of Damage	
Medical Severity	0.015	<0.01
Serious dependence (EIDA)	0.018	<0.01
Total EIDA score	0.006	<0.01
	Gender—Reward Dependency	
Family Severity	0.047	<0.05
	Gender—SD	
Obsessive component score (OCDS)	0.002	<0.01
Total OCDS score	0.024	<0.05
	Gender—Self-Autotranscende	
Psychiatric Severity	0.005	<0.01
